# Improving Doctors’ Confidence in Referral Pathways at a Stand-Alone Mental Health Hospital: A Quality Improvement Project

**DOI:** 10.1192/bjo.2026.11477

**Published:** 2026-06-30

**Authors:** Lisa Schomerus, Subhiksha Loganathan, Kathryn McCabe, Georgy Pius

**Affiliations:** Greater Manchester Mental Health, Manchester, United Kingdom

## Abstract

**Aims:**

Doctors working in stand-alone mental health hospitals frequently need to refer patients to acute and specialist services to manage physical health problems. These referrals can involve multiple acute hospital trusts, resulting in unclear and inconsistent referral pathways. Resident doctors who rotate regularly between placements may in particular be unfamiliar with local pathways, which can lead to inefficiencies and impact quality of patient care. The aim of this quality improvement project was to assess and improve doctors’ confidence in making appropriate referral pathways by developing and implementing a referral guidance resource. It was hypothesised that compiling a comprehensive referral guidance resource would improve doctors’ self-reported confidence.

**Methods:**

This quality improvement project was carried out at Atherleigh Park Hospital using two Plan–Do–Study–Act cycles. Two cohorts of doctors participated (Cycle 1: n=10; Cycle 2: n=6). Baseline confidence in identifying referral pathways was assessed using a questionnaire with a five-point Likert scale (1=not at all confident, 5=very confident). A referral guidance resource outlining commonly required specialty referral pathways was developed and implemented. Following implementation, participants completed a repeat questionnaire assessing confidence, resource usage, ease of access, and included opportunity for qualitative feedback.

**Results:**

Baseline confidence varied across both cohorts. Following the introduction of the referral guidance resource, 100% of respondents reported having used the resource whilst 94% reported knowing where to find it. All respondents also reported an improvement in confidence in identifying appropriate referral pathways. Post-intervention confidence scores across both cohorts ranged from 4 to 5, with a mean score of 4.5 out of 5. Ongoinguncertainty in specific specialties, such as referrals to respiratory medicine, venous thromboembolism clinic, and referrals outside the main acute hospital trust affiliated with the mental health hospital were identified through qualitative feedback. The findings have informed priorities for further development of the resource.

**Conclusion:**

The introduction of a referral guidance resource was associated with improved self-reported confidence. There was high uptake of the resource among doctors at a stand-alone mental health hospital. The findings support the hypothesis that a structured referral resource can improve clinician confidence and show the capacity to contribute to meaningful local service improvement. Further work will focus on expanding specialty coverage within the resource, which has already been incorporated into the resident doctor induction sessions at the hospital with the aim to expand its impact.

